# The Combination Effect of *Purpureocillium lilacinum* Strain (AUMC 10620) and Avermectin (B1a and B1b) on Control Citrus Nematode *Tylenchulus semipenetrans* (Cobb) Under Laboratory and Field Conditions

**DOI:** 10.3390/biology14010060

**Published:** 2025-01-13

**Authors:** Amr M. El-Marzoky, Mohamed A. M. S. Ali, Ahmed S. M. Elnahal, Dalia A. Abuljadayel, Wafa A. H. Alkherb, Mahmoud Moustafa, Mohammed O. Alshaharni, Elsayed M. Abd El-Aal

**Affiliations:** 1 Plant Protection Department, Faculty of Agriculture, Zagazig University, Zagazig 44511, Egypt; amelmarzoky@agri.zu.edu.eg (A.M.E.-M.);; 2 Plant Pathology Department, Faculty of Agriculture, Zagazig University, Zagazig 44511, Egypt; melderiby@zu.edu.eg; 3 Department of Biological Sciences, Faculty of Science, Zoology, King Abdulaziz University, Jeddah 21461, Saudi Arabia; dabuljadayl@kau.edu.sa; 4 Department of Biology, College of Science, Qassim University, Buraidah 1452, Saudi Arabia; 5 Department of Biology, College of Science, King Khalid University, Abha 9004, Saudi Arabiamaasalim@kku.edu.sa (M.O.A.)

**Keywords:** biocontrol, citrus nematodes, *Tylenchulus semipenetrans*, *Purpureocillium lilacinum*, avermectin, PPNs, integrated pest management, eco-friendly pest control

## Abstract

This study investigates the nematicidal efficacy of *Purpureocillium lilacinum* strain (AUMC 10620) combined with avermectin (B1a and B1b) against the citrus nematode *Tylenchulus semipenetrans*, both in vitro and in vivo. Laboratory experiments tested four concentrations of *P. lilacinum* (2.5, 5, 10, and 15 × 10^7^ spores/mL) mixed with 250 ppm avermectin, assessing their effects on nematode juveniles (J2) and eggs. Results showed significant increases in juvenile mortality and egg hatching inhibition with the combined treatments compared to individual applications. The highest concentration (15 × 10^7^ spores/mL + 250 ppm avermectin) achieved 100% juvenile mortality and 83.33% egg hatching inhibition after 72 h. Field trials confirmed the superior efficacy of the combined treatment, with *P. lilacinum* (15 × 10^7^ spores/mL) and avermectin (1000 ppm) reducing nematode populations by 70.85% in mandarin trees and 70.77% in sweet orange trees after three weeks, compared to the 83.59% and 81.76% reduction, respectively, achieved by oxamyl. These findings suggest that mixtures of *P. lilacinum* and avermectin can serve as effective, eco-friendly alternatives for controlling nematodes in citrus orchards.

## Simple Summary

This study explores the combined effect of *P. lilacinum*, a fungus, and avermectin, a pesticide, on controlling citrus nematodes (*T. semipenetrans*), a pest that damages citrus crops. The research found that the combination was more effective than using either treatment alone, both in the lab and in the field. The combined treatment reduced nematode numbers and reproduction more significantly over time. These results suggest that using both treatments together could help improve pest control and protect citrus crops, offering a more sustainable solution for farmers.

## 1. Introduction

Plant-parasitic nematodes (PPNs) are significant pathogens affecting citrus worldwide, causing about 10–30% yield loss due to infection by these soil-borne pathogens [1]. *Tylenchulus semipenetrans* is one of the most damaging nematodes in citrus orchards [2,3], responsible for the disease known as citrus dieback, which results in 15–35% crop yield losses [4]. Globally, annual losses due to this nematode infestation are estimated between 8.7% and 12.2%, with potential increases up to 14% [5,6].

In recent years, biocontrol agents have gained popularity for managing agricultural pests, particularly PPNs. Among these, *Purpureocillium lilacinum* has emerged as a promising biocontrol agent. The genus *Purpureocillium* is recognized as one of the most efficient antagonists for PPNs [7]. In addition, *P. lilacinum* (Thom) Samson, formerly known as *Paecilomyces lilacinus*, is a saprophytic soil fungus and a critical egg-pathogenic agent against cyst and root-knot nematodes [8]. This fungus can damage nematodes by colonizing their cuticle, with its hyphae either directly penetrating the cuticle and eggshell or releasing hydrolytic enzymes like proteases and chitinases [9].

Isaaca et al. [10] used the fungus *P. lilacinum* native strain (AUMC 10149) in a concentration (1 × 10^8^ CFU/mL) of 10 mL/pot planted with tomatoes. They indicated that it reduced the J2 of *M*. *incognita* by 97.6% and egg hatching by 79.8% after 72 h of exposure. Leong et al. [11] found a highly significant infection of >90% at *p* ≤ 0.01 on *M. incognita* female nematodes when they tested the nematicidal properties of ten Malaysiaian native strains of *P. lilacinum* compared to a commercial strain on different stages of *M. incognita*. They indicated that, at a concentration of 10^5^ spore/mL, the parasitism on the eggs varied from 66.0 to 78.8%, and the egg hatching inhibition reached 89% after seven days of exposure.

Avermectin is a major metabolite of the bacteria *Streptomyces avermitilis*; it has a nematicidal effect on a wide range of PPNs. It is the main compound in the nematicide abamectin [12,13]. Abamectin is part of the avermectin group, composed of lactone macrocyclic metabolites produced during the natural fermentation process of the bacterium *S. avermitilis* [13]. This bacterium contains about 80% avermectin B1a and 20 percent avermectin B1b [14]. Abamectin is very effective in controlling plant-parasitic nematodes, with no harmful effect on seed germination and plant growth [15,16].

This study aims to evaluate the nematicidal efficacy of a *P. lilacinum* strain (AUMC 10620) and avermectin (B1a and B1b), both individually and in combination, against the citrus nematode *T. semipenetrans* in laboratory and field conditions. It focuses on determining the optimal concentrations for maximum juvenile mortality and egg hatching inhibition in vitro and assesses the impact of combined treatments on nematode populations in mandarin and sweet orange trees in the field. Additionally, this study compares these treatments with the standard nematicide oxamyl to explore the potential of *P. lilacinum* and avermectin mixtures as eco-friendly alternatives for nematode control in citrus orchards.

## 2. Materials and Methods

### 2.1. Experimental Design

This study was conducted both in the laboratory and in the field. Soil and root samples were collected from 15-year-old sweet orange trees (*Citrus sinensis*) grafted onto sour orange rootstock (*C. aurantium*) in a six-feddan area. Samples were taken from a depth of 20–25 cm and at a distance of 1.5 m from the tree trunk. A total of 10 samples were randomly collected, placed in polyethylene bags, and transported to the nematode laboratory at Zagazig University, Egypt. The samples were stored at 5 °C and prepared for extraction the following day. The samples were then combined into a composite sample of approximately 1 kg [17].

### 2.2. Extraction of Citrus Nematode Second-Stage Juveniles (J2)

The second-stage juveniles (J2) of *T. semipenetrans* were extracted from soil samples using sieves and Baermann trays [18,19]. One mL of nematode suspension was pipetted onto a Hawksley counting slide for morphological identification and counting under a microscope at 100× magnification [20]. One mL of the suspension containing about 500 J2 was added to each 15 cm diameter Petri dish with a medium of 7.5 g agar in 1 L of distilled water, with 5 mL of 25% lactic acid added to prevent bacterial growth. Each treatment was replicated three times. The control contained only the J2 suspension and distilled water, while other dishes contained nematodes and the tested materials. Mortality of the juveniles (immobile and straight) was recorded at 24, 48, and 72 h. The percent mortality was calculated using Abbott’s formula [21].

(1) $$\mathrm{Percent}\;\mathrm{of}\;\mathrm{correct}\;\mathrm{mortality}\;(\%)=\frac{Treat\;mentmortality\left(\%\right)-Control\;mortality\left(\%\right)}{100-Mortality\;in\;control}\times 100$$

The synergy factor (SF) was calculated according to the Colby formula [21,22]. 

The expected response *E* of the combination (*E Colby*) = *P*_1_ *+ P*_2_ *−* $\frac{P_{1}P_{2}}{100}$.

(2) $$\mathrm{SF}=\frac{E\;measured}{E\;Colby}$$

where *P*_1_ and *P*_2_ are the respective efficacies of the two compounds (in %); by efficacy we mean the percentage of inhibition. (*E *_measured_) is the observed experimental efficacy of the mixture.

### 2.3. Preparation of Root Samples for Nematode Egg Extraction

To extract nematode eggs, a sodium hypochlorite solution was used to dissolve the gelatinous matrix surrounding the eggs. Following Hussey and Barker [23], 20 mL of 5% sodium hypochlorite (Clorox^®^, Oakland, CA, USA) was mixed with 180 mL of distilled water to make a 0.5% solution. Ten grams of citrus roots were soaked in water for five minutes, cut into 2 cm pieces, washed with tap water, and placed in a 500 mL flask with 200 mL of the sodium hypochlorite solution. The roots were shaken for about 3 min to isolate the eggs. The suspension was poured through a 200 mesh sieve over a 500 mesh sieve. The lower sieve contents were rinsed with tap water to remove sodium hypochlorite residues, ensuring egg viability. The eggs were then transferred to a 100 mL beaker, and their number per milliliter was counted using a microscope.

Around 500 citrus nematode eggs (1 mL of suspension) were added to each 15 cm Petri dish containing the prepared medium to allow fungal spore growth and egg parasitism. Each treatment was replicated three times, with dishes incubated at 25 ± 2 °C and 75% humidity. Egg hatching rates were recorded at 24, 48, and 72 h, calculated according to Sun et al. [24].

(3) $$\mathrm{The}\;\mathrm{eggs}\;\mathrm{hatching}\;\mathrm{rate}=\frac{Juveniles}{Initial\;number\;of\;eggs}\times 100$$

The ovicidal efficacy of the tested materials was determined by calculating the egg hatching inhibition percentage in Equation (4).

(4) $$\mathrm{The}\;\mathrm{inhibition}\;\mathrm{in}\;\mathrm{eggs}\;\mathrm{hatching}=\frac{The\;initial\;number\;of\;the\;eggs-Number\;of\;hatched\;eggs}{The\;initial\;number\;of\;the\;eggs}\times 100$$

### 2.4. Preparation of Avermectin and P. lilacinum for Testing on J2 and Eggs

Avermectin, sourced from the commercial product Tervigo^®^ 2% SC (Syngenta Agrochemical Company, Basel, Switzerland), which contains 80% avermectin B1a and 20% avermectin B1b, was used in this study. To create a 250 ppm solution, the formulation bottle was thoroughly shaken, and 12.5 mL was measured into a 1000 mL flask, then diluted to 1 L with distilled water—this concentration represents one quarter of the recommended dose. Both the citrus nematode J2 and eggs were treated with this solution, either alone or mixed with various concentrations of *P. lilacinum* to assess their compatibility.

For the field experiments, a 1000 ppm solution (the recommended dose) was prepared by mixing 50 mL of Tervigo^®^ in 1 L of distilled water [25].

The *P. lilacinum* strain (AUMC 10620) from Zagazig University’s Plant Pathology Department was cultured on barley grains inoculated with conidia from seven-day-old plates. After three weeks at 25 ± 1 °C, 500 mg of culture was mixed into 30 mL of 0.05% sterile agar solution (agar powder, C_14_H_24_O_9_, 1.5% concentration, Manufacturer Hebei Zhuanglai Chemical Trading Co., Ltd. Shijiazhuang, China, CAS number 9002-18-0). The conidia count per mL was measured using a hemocytometer.

A standard fungal solution (15 × 10^7^ spores/mL) was prepared. Serial dilutions (2.5, 5, and 10 × 10^7^ spores/mL) were created using the formula provided by Castaño-Zapata [26]:

(5) Initial concentration of spores/mL=(The final volume of the suspension×Initial volume of the suspension)/(The final concentration of spores/mL)

One mL of each spore suspension (5, 10, and 15 × 10^7^ spores/mL) was mixed with one mL of 250 ppm avermectin and added to sterile 10 cm diameter Petri dishes containing an agar medium at a 1.5% concentration. The J2 and eggs were placed separately in each dish, and data were recorded at specified intervals.

### 2.5. The Field Experiment

A field study was conducted to verify the laboratory results on a six-feddan loamy soil area, divided into two sections: one section with 15-year-old mandarin trees (*C. reticulata*) and the other with 12-year-old sweet orange trees (*C. sinensis*), both located in Abu-Hammad, Al-Sharkia Governorate, Egypt (30°27′58.6″ N 31°40′04.6″ E). Each section was arranged in five rows with different treatments: oxamyl (Vydate^®^ 10% G) at 150 g/tree, avermectin at 1000 ppm, *P. lilacinum* at 15 × 10^7^ spores/mL, and a combination of avermectin and *P. lilacinum*. Each tree received a 100 mL injection into the upper 30 cm layer of the soil around the rhizosphere for the respective treatments. The control group received no treatment. Soil samples were taken one, two, and three weeks post-treatment to count active *T. semipenetrans* J2. Nematode reduction was calculated using the following Formula (6):

(6) $$\mathrm{Nematode}\;\mathrm{reduction}\;\mathrm{percentage}\;(\%)=\frac{Number\;of\;nematodes\;in\;the\;Control-Number\;of\;nematodes\;in\;the\;treatment}{Number\;of\;nematodes\;in\;the\;Control}\times 100$$

### 2.6. Statistic Analysis

The data were analyzed using SPSS version 16 and version 9.4 (Cary, NC, USA). For the laboratory experiments, a two-way ANOVA was employed to analyze the effects of treatment and exposure time, with post-hoc tests and LSD calculations at *p* ≤ 0.05 to assess significant differences among treatments. For the field data, we applied a pseudo-replication methodology using SAS as described by Esparza-Diaz et al. [27].

## 3. Results

### 3.1. Efficacy of the Avermectin and P. lilacinum Mixture on Citrus Nematode (J2) In Vitro

The effects of the combination of *P. lilacinum* and avermectin on *T. semipenetrans* J2 (juvenile nematodes) are presented in Table 1. After 24 h, the most effective treatment was the combination of *P. lilacinum* (15 × 10^7^ spores/mL) and avermectin, resulting in 373.33 dead J2, which significantly exceeded the control group (35 dead J2), avermectin alone resulted in 145 dead J2, and *P. lilacinum* alone in 152 dead J2. The second most effective treatment was *P. lilacinum* (10 × 10^7^ spores/mL) combined with avermectin, which resulted in 340.00 dead J2, followed by *P. lilacinum* (5 × 10^7^ spores/mL) plus avermectin with 228.33 dead J2.

At 48 h, the mortality rates increased across all treatments. The combination of *P. lilacinum* (15 × 10^7^ spores/mL) and avermectin showed the highest mortality, with 493.33 dead J2, followed closely by *P. lilacinum* (10 × 10^7^ spores/mL) plus avermectin (466.67 dead J2) and *P. lilacinum* (5 × 10^7^ spores/mL) plus avermectin (460.00 dead J2). In comparison, the control, *P. lilacinum* (2.5 × 10^7^ spores/mL), and avermectin alone showed much lower mortality, with 235.33, 225.00, and 118.33 dead J2, respectively. No significant differences were found between avermectin 250 ppm with *P. lilacinum* (2.5 × 10^7^ spores/mL) and the combinations of *P. lilacinum* (5 × 10^7^ spores/mL) plus avermectin and *P. lilacinum* (10 × 10^7^ spores/mL) plus avermectin.

At 72 h, *P. lilacinum* (15 × 10^7^ spores/mL) plus avermectin remained the most effective treatment, achieving 500.00 dead J2 compared to 150.00 in the control, 280.00 with avermectin alone, and 293.33 with *P. lilacinum* (2.5 × 10^7^ spores/mL). Table 2 summarizes the cumulative mortality rates, with the highest mortality observed for *P. lilacinum* (15 × 10^7^ spores/mL) plus 250 ppm avermectin at 72 h, reaching 100.00%. The mortality rates for other combinations, such as *P. lilacinum* (5 × 10^7^ spores/mL) plus avermectin and *P. lilacinum* (10 × 10^7^ spores/mL) plus avermectin, were also high, with final mortality rates of 91.42% and 93.33%, respectively.

In comparison, avermectin alone achieved mortality rates of 23.65%, 27.95%, and 37.14% at 24, 48, and 72 h, respectively, while *P. lilacinum* (2.5 × 10^7^ spores/mL) resulted in 25.48%, 30.65%, and 40.95% mortality, respectively. These results confirm the superior efficacy of the combined treatment of *P. lilacinum* and avermectin in controlling *T. semipenetrans* J2 in vitro.

### 3.2. The Effect of the Tested Mixtures on the Egg Hatching of Citrus Nematodes In Vitro

The effects of the tested mixtures on the egg hatching are presented in Table 2. Significant differences were observed between avermectin 250 ppm and *P. lilacinum* (2.5 × 10^7^ spores/mL) in comparison with the control treatment after 24 h of exposure. The number of hatched eggs was 213.33 in the control, 201.33 in *P. lilacinum* (2.5 × 10^7^ spores/mL), and 215.00 in avermectin, which were higher compared to the combined treatments, which showed significantly reduced hatching: 105.00, 140.00, and 170.00 for *P. lilacinum* (15 × 10^7^ spores/mL) + avermectin, *P. lilacinum* (10 × 10^7^ spores/mL) + avermectin, and *P. lilacinum* (5 × 10^7^ spores/mL) + avermectin, respectively.

After 48 h, the suppression of egg hatching increased in all treatments. The number of hatched eggs decreased significantly in *P. lilacinum* (15 × 10^7^ spores/mL) + avermectin, with 98.33 fewer eggs compared to the control, which had 218.33 hatched eggs. The other treatments, *P. lilacinum* (10 × 10^7^ spores/mL) + avermectin and *P. lilacinum* (5 × 10^7^ spores/mL) + avermectin, showed 125.00 and 165.00 hatched eggs, respectively. In contrast, avermectin (250 ppm) and *P. lilacinum* (2.5 × 10^7^ spores/mL) resulted in 200.00 and 198.33 hatched eggs, respectively.

The most significant suppression of egg hatching was observed at 72 h. The combined treatment of *P. lilacinum* (15 × 10^7^ spores/mL) + avermectin showed the highest reduction, with only 83.33 eggs hatching. The treatments *P. lilacinum* (10 × 10^7^ spores/mL) + avermectin and *P. lilacinum* (5 × 10^7^ spores/mL) + avermectin followed with 98.33 and 150.00 hatched eggs, respectively. In comparison, the number of hatched eggs was 190.00 for *P. lilacinum* (2.5 × 10^7^ spores/mL) and 198.33 for avermectin alone. The control treatment had the highest hatching rate, with 270.00 eggs hatching.

The egg hatching rates were calculated to quantify the effect of the tested mixtures on egg hatching (Table 2). After 24 h of exposure, the treatments were arranged in descending order as follows: *P. lilacinum* (15 × 10^7^ spores/mL) + avermectin, *P. lilacinum* (10 × 10^7^ spores/mL) + avermectin, *P. lilacinum* (5 × 10^7^ spores/mL) + avermectin, *P. lilacinum* (2.5 × 10^7^ spores/mL), and avermectin (250 ppm). The egg hatching rates were 21.00%, 28.00%, 34.00%, 40.26%, and 42.66%, respectively, while the control treatment had a hatching rate of 43.00%. The most effective combination after 48 h was *P. lilacinum* (15 × 10^7^ spores/mL) + avermectin, which reduced the hatching rate to 19.66% compared to the control (43.66%). After 72 h, the hatching rate was reduced to 16.66% in *P. lilacinum* (15 × 10^7^ spores/mL) + avermectin, while the control had a hatching rate of 54.00%.

**Figure 1:**
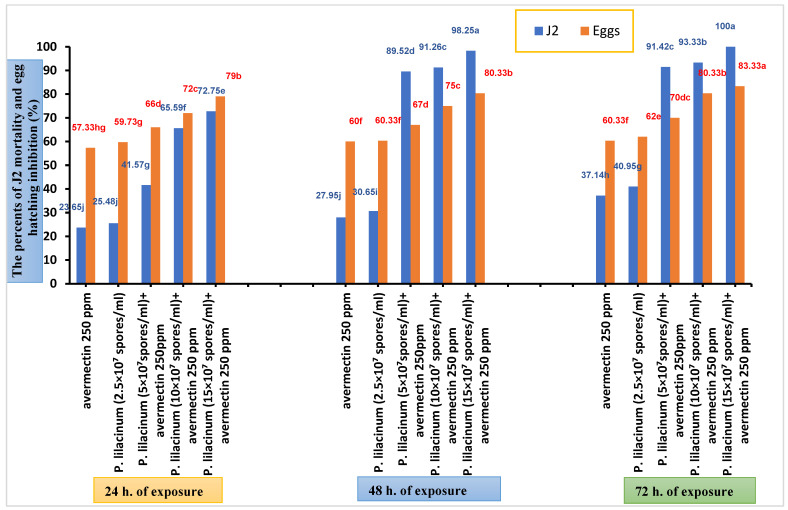
The effect of the tested material on citrus nematode (J2) mortality and egg hatching inhibition after 24, 48, and 72 h of the treatment. Percentages in the same-colored column followed by the same letter are not significantly different according to the LSD test (*p* ≤ 0.05).

In conclusion, the combination treatments, particularly *P. lilacinum* (15 × 10^7^ spores/mL) + avermectin, were the most effective in suppressing egg hatching and inducing mortality in J2 nematodes when compared to the control treatment over all three exposure periods (Figure 1).

Data showed that, after 24 h of treatment, the highest effect on both J2 mortality and egg hatching inhibition was observed with the three mixtures. The most significant inhibition was 79.00% in the *P. lilacinum* (15 × 10^7^ spores/mL) + avermectin treatment, while the lowest effect on J2 was recorded at 72.75% in the same treatment. However, after 48 and 72 h of exposure, the impact shifted, with the mixtures showing a stronger effect on J2 than on egg hatching. The highest effect on J2 mortality was observed in the *P. lilacinum* (15 × 10^7^ spores/mL) + avermectin treatment, reaching 100% after 72 h and 98.25% after 48 h. In contrast, the inhibition of egg hatching in this treatment was 83.33% after 72 h and 80.33% after 48 h. These results indicate that the tested combinations were most effective after 48 h, with their efficacy peaking on J2 nematodes rather than eggs.

### 3.3. The Efficacy of Combination Treatment (Avermectin and P. lilacinum) Against T. semipenetrans Under Field Conditions

The nematocidal efficacy of the combination of *P. lilacinum* and avermectin was further tested under field conditions to validate the results obtained in vitro. The experiment was conducted on two plant hosts, and the data for mandarin trees are presented in Table 3. Significant differences were observed between all treatments and the control group.

Oxamyl was the most effective treatment, reducing J2 numbers to 1163.4, 802.00, and 405.4 J2/250 g soil after one, two, and three weeks of treatment, respectively, with corresponding reduction percentages of 50.77%, 66.96%, and 83.59%. In contrast, the combined treatment of *P. lilacinum* and avermectin resulted in J2 numbers of 1623.4, 1127.8, and 720.40 J2/250 g soil, with reduction percentages of 31.31%, 53.55%, and 70.85% at the same time intervals. The J2 number in the control treatment was 2363.40, 2427.8, and 2471.40 J2/250 g soil after one, two, and three weeks, respectively.

The J2 number in the separate treatments of avermectin and *P. lilacinum* was significantly higher compared to the combined treatment. For instance, after three weeks, the J2 number in the avermectin treatment was 1327.40 J2/250 g of soil, with a reduction of 46.29%. In the *P. lilacinum* treatment, the J2 number was 1050.00 J2/250 g of soil, corresponding to a reduction of 57.51%.

A similar trend was observed in sweet orange trees (Table 4). Oxamyl, again, proved to be the most effective treatment for reducing J2 numbers, followed by the combined *P. lilacinum* and avermectin treatment. After one week of application, J2 numbers decreased from 2466.40 in the control to 1227.40 in the oxamyl treatment and 1655.40 in the combined treatment, with reduction percentages of 50.33% and 32.88%, respectively. There were no significant differences between the avermectin and *P. lilacinum* treatments and the control. The J_2_ number was 2148.40, 2014.40, and 2466.40 J2 in 250 g/soil consecutively after one week of application.

The lethal effect of the treatments increased over time. After two weeks, the J2 number in 250 g of soil was 866.00 in the oxamyl treatment and 1159.80 in the combined treatment, while the J2 number in the avermectin and *P. lilacinum* treatments was 1670.80 and 1428.80, respectively, compared to 2530.80 in the control. The reduction percentages were as follows: oxamyl (65.78%), *P. lilacinum* + avermectin (54.14%), *P. lilacinum* (43.54%), and avermectin (33.98%).

The maximum effect was observed after three weeks of treatment. Oxamyl reduced the J2 number from 2574.40 to 469.40, with a reduction percentage of 81.76%. The combined treatment reduced the J2 number to 752.40, with a reduction of 70.77%. The *P. lilacinum* treatment achieved a reduction of 57.23%, while the avermectin treatment reduced the J2 number by 46.76%.

In conclusion, the results support the use of the combination of *P. lilacinum* and avermectin over their separate applications for controlling *T. semipenetrans* under both laboratory and field conditions. These findings highlight the potential benefits of mixing these biocontrol agents and their incorporation into integrated pest management programs.

## 4. Discussion

The results obtained in this research paper show the efficacy of mixing fungal spores of *P. lilacinum* and *S. avermitilis* metabolite (avermectin) as nematode biocontrol agents to enhance their efficacy rather than using each one alone in controlling larval and egg stages of citrus nematode in vitro and in vivo. All the tested mixtures had a powerful lethal effect on J_2_ and eggs compared to each separately under laboratory conditions. This effect was confirmed under field conditions; therefore, the combined treatment was more effective than the separate treatments. These results push to confirm the hypothesis that mixtures of some microbial biocontrol agents may be more effective in reducing the number of pests [28].

The efficacy of *P. lilacinum* as a biocontrol agent has been widely reported. For example, Kepenekci et al. [29] found that different concentrations of *P. lilacinum* significantly reduced nematode reproduction in *Pratylenchus thornei*, a root-lesion nematode that affects wheat. They applied the fungus at 10^6^, 10^7^, and 10^8^ conidia cultures mL. It was found that all fungal concentrations decreased nematode reproduction. Sarven et al. [30] showed that applying the soil with *P. lilacinum* at 1 × 10^6^ CFU/g soil before and after three days of eggplant transplantation decreased the root gall index by 72% and the egg mass by 84% for *M. incognita*. These findings align with our results, where the fungal agent alone proved effective against *T. semipenetrans*, but its combination with avermectin significantly enhanced its nematicidal activity.

Sharma et al. [31] tested the *P. lilacinus* formulation based on Karanja deoiled cake and sundried biogas slurry and its ability to colonize a root-knot egg mass. They indicated that the test-based formulation was more efficient in controlling *M. incognita* than the formulation based on the traditional substrate wheat. The egg mass inhibition reached 96.8% and a superior colonization ability (100% colonization of egg mass on the third day).

Regarding the effect of avermectin on PPNs, several studies have confirmed the efficacy of this material, e.g., El-shahaat et al. [32] compared the abamectin product (Tervigo^®^) with other treatments to manage *T. semipenetrans* in Washington navel orange trees (*C. sinensis*) in Egypt. Nematodes were reduced by 78–87% after the application, comparable with oxamyl and azadirachtin. El-Saedy et al. [33] tested the nematicidal efficacy of five eco-friendly materials, including abamectin, compared to oxamyl 24% SL against the citrus nematode *T. semipenetrans*. The results indicated that abamectin has a potent nematicidal effect and can be used in PPN controlling programs. Khalil and Darwesh [34] reported that avermectin B1 has efficacy in managing a wide range of pests, including PPNs. This nematicide can be applied through many different methods, such as soil treatment, seed treatment, injection into plant stem, and seedling root dip.

Sasanelli et al. [13] decided that soil application of abamectin significantly reduced the cyst nematode *Globodera pallida* in potato crops, and they recommended using abamectin in organic agriculture or integrated pest management programs. Sasanelli et al. [35] reported that avermectin had nematicidal properties against some PPNs such as *M. incognita*, *Pratylenchus penetrans*, *Rotylenchulus reniformis*, *G. pallida*, *Heterodera schachtii*, *H. avenae*, and *H. carotae*. Its mode of action could be summarized as blocking γ-aminobutyric acid by stimulating chloride channels, leading to the opening of chloride channels, resulting in nematode paralysis. El-Marzoky et al. [16] indicated that abamectin (avermectin) had a potential effect on controlling *M. incognita* when used as cucumber seed treatment before planting, with no adverse impact on seed germination and vegetative parameters.

In addition, the synergistic effect of combining biocontrol agents is well documented. El-Ashry et al. [36] studied the effect of four biocontrol agents and three botanical extracts individually and in three combinations against *M. incognita*. They showed that combining *P. lilacinum* and avermectin effectively reduced root galls and nematode reproduction of *M. incognita*, while also improving plant growth parameters. Similarly, Khairy et al. [37] tested the nematicidal efficacy of abamectin and *P. lilacinum* separately or combined with moringa extract on *M.-incognita*-infected eggplants. They found that all the combined treatments were more effective than the single treatments. At the end of the experiment, the results indicated that the biocontrol agent’s efficacy was enhanced by the mixing with moringa extracts. All nematode parameters were decreased compared to the control treatment, and there were no significant differences between these mixtures and oxamyl. Moreover, Kumar and Arthurs [38] indicated that citrus groves infested with many plant-parasitic nematode species, including *T. semipenetrans*, *Radopholus similis*, *Pratylenchus coffeae*, and *Meloidogyne* spp., and various microbial agents can use the following to control these PPNs: e.g., fungi (*Trichoderma* spp., *P. lilacinum*, *Pochonia chlamydosporia*, and mycorrhizae, *Glomus* spp.) and bacteria like *Bacillus* spp., *P. fluorescens*, *S. avermitilis*, and *Pasteuria* spp. These biocontrol agents enhance the control strategy when used together or with the other control methods.

## 5. Conclusions

This study demonstrates that combining a *P. lilacinum* strain (AUMC 10620) with avermectin (B1a and B1b) significantly enhances the control of the citrus nematode *T. semipenetrans* compared to using each agent individually. The combined treatments achieved higher juvenile mortality and egg hatching inhibition in vitro, particularly at the highest tested concentration (15 × 10^7^ spores/mL + 250 ppm avermectin). Field trials further confirmed these results, showing that the combined treatment significantly reduced nematode populations in mandarin and sweet orange trees, achieving reduction percentages comparable to those achieved by the chemical nematicide oxamyl. These results suggest that the combination of *P. lilacinum* and avermectin could be a viable, eco-friendly alternative for managing citrus nematodes in orchards, promoting sustainable agricultural practices for integrated pest management (IPM). Further research is needed to evaluate the long-term effects of this combination in field conditions, assess its impact on non-target organisms, and explore its compatibility with other pest management strategies.

## Figures and Tables

**Table 1:** The lethal effect of different concentrations of *P. lilacinum* spores and avermectin on *T. semipenetrans* juveniles in vitro after 24, 48, and 72 h of application.

Treatments	No. of Dead J2 After 24 h	No. of Dead J2 After 48 h	No. of Dead J2 After 72 h
Negative control (nematodes + distilled water)	35.00 e ± 2.88	118.33 d ± 4.41	150.00 d ± 2.88
Mortality percentage (%)	0	0	0
Nematodes + avermectin 250 ppm	145.00 d ± 2.88	225.00 c ± 2.88	280.00 c ± 5.77
Mortality percentage (%)	23.65	27.95	37.14
Nematodes + *P. lilacinum* (2.5 × 10^7^ spores/mL)	152.00 d ± 2.78	235.33 c ± 5.68	293.33 c ± 3.77
Mortality percentage (%)	25.48	30.65	40.95
Nematodes + *P. lilacinum* (5 × 10^7^spores/mL) + avermectin 250 ppm	228.33 c ± 4.41	460.00 ab ± 23.09	470.00 ab ±2 0.81
Mortality percentage (%) (SF)	41.57 (0.96)	89.52 (1.78)	91.42 (1.45)
Nematodes + *P. lilacinum* (10 × 10^7^ spores/mL) + avermectin 250 ppm	340.00 b ± 5.77	466.67 ab ± 14.53	476.67 ab ± 17.63
Mortality percentage (%) (SF)	65.59 (1.52)	91.26 (1.82)	93.33 (1.48)
Nematodes + *P. lilacinum* (15 × 10^7^ spores/mL) + avermectin 250 ppm	373.33 a ± 18.55	493.33 a ± 3.33	500.00 a ± 0.00
Mortality percentage (%) (SF)	72.75 (1.68)	98.25 (1.96)	100.00 (1.59)

Means in the same column followed by the same letter are not significantly different according to the LSD test (*p* ≤ 0.05). Values between parentheses refer to the ratio of synergy factor (SF). If SF > 1, synergism; if SF < 1, antagonism.

**Table 2:** The lethal effect of different concentrations of *P. lilacinum* spores and avermectin on *T. semipenetrans* eggs hatching in vitro after 24, 48, and 72 h of application.

Treatments	No. of Hatched Eggs After 24 h	No. of Hatched Eggs After 48 h	No. of Hatched Eggs After 72 h
Negative control (Nematode eggs + distilled water)	215.00 a ± 2.88	218.33 a ± 4.41	270.00 a ± 5.77
Egg hatching rate (%)	43.00	43.66	54.00
Nematode eggs + avermectin 250 ppm	213.33 b ± 17.63	200.00 b ± 5.77	198.33 a ± 6.00
Egg hatching rate (%)	42.66	40.00	39.66
Nematode eggs + *P. lilacinum* (2.5 × 10^7^ spores/ml	201.33 b ± 7.63	198.33 b ± 7.00	190.00 a ± 4.33
Egg hatching rate (%)	40.26	39.66	38.00
Nematode eggs + *P. lilacinum* (5 × 10^7^spores/mL) + avermectin 250ppm	170.00 c ± 5.77	165.00 c ± 2.88	150.00 b ± 5.77
Egg hatching rate (%)	34.00	33.00	30.00
Nematode eggs + *P. lilacinum* (10 × 10^7^ spores/mL) + avermectin 250 ppm	140.00 cd ± 5.77	125.00 d ± 2.88	98.33 c ± 4.41
Egg hatching rate (%)	28.00	25.00	19.66
Nematode eggs + *P. lilacinum* (15 × 10^7^ spores/mL) + avermectin 250 ppm	105.00 e ± 2.88	98.33 e ± 6.00	83.33 cd ± 4.41
Egg hatching rate (%)	21.00	19.66	16.66

Means in the same column followed by the same letter are not significantly different according to the LSD test (*p* ≤ 0.05).

**Table 3:** The effect of avermectin and *P. lilacinum* in separate and combined applications on *T. semipenetrans* infesting a mandarin orchard in a mixture and separate after one, two, and three weeks of the treatment.

Treatments	Number of J2 in 250 g/Soil		
	One Week After Treatment	Two Weeks After Treatment	Three Weeks After Treatment
Control	2363.40 a	2427.80 a	2471.40 a
	(0%)	(0%)	(0%)
Oxamyl (10% G)	1163.40 e	802.00 e	405.40 e
	(50.77%)	(66.96%)	(83.59%)
Avermectin (1000 ppm)	2105.40 a	1627.80 b	1327.40 b
	(10.92%)	(32.95%)	(46.29%)
*P. lilacinum* (15 × 10^7^ spores/mL)	1963.40 ab	1377.80 c	1050.00 c
	(16.92%)	(43.24%)	(57.51%)
Avermectin + *P. lilacinum* (in the abovementioned concentrations)	1623.40 d	1127.80 d	720.40 d
	(31.31%)	(53.55%)	(70.85%)

The means followed by the dissimilar letter (s) in the same column varied significantly according to the LSD test (*p* ≤ 0.05). Values between parentheses indicate the reduction percentages (%) according to Formula (6).

**Table 4:** The effect of the avermectin and *P. lilacinum* in separate and combined applications on *T. semipenetrans* infesting a sweet orange orchard in the form of a mixture and separate after one, two, and three weeks of the treatment.

Treatments	Number of J2 in 250 g/Soil		
	One Week After Treatment	Two Weeks After Treatment	Three Weeks After Treatment
Control	2466.40 a	2530.8 a	2574.40 a
	(0%)	(0%)	(0%)
Oxamyl (10% G)	1227.40 e	866.00 e	469.40 e
	(50.33%)	(65.78%)	(81.76%)
Avermectin (1000 ppm)	2148.40 a	1670.80 b	1370.40 b
	(12.89%)	(33.98%)	(46.76%)
*P. lilacinum* (15 × 10^7^ spores/mL)	2014.40 ab	1428.80 c	1101.00 c
	(18.33%)	(43.54%)	(57.23%)
Avermectin + *P. lilacinum* (in the abovementioned concentrations)	1655.40 d	1159.80 d	752.40 d
	(32.88%)	(54.17%)	(70.77%)

The means followed by the dissimilar letter (s) in the same column varied significantly according to the LSD test (*p* ≤ 0.05). Values between parentheses indicate the reduction percentages (%) according to Formula (6).

## Data Availability

All data were included within this article, and any additional information could be provided by the corresponding author.
